# More Teeth and Posterior Balanced Occlusion Are a Key Determinant for Cognitive Function in the Elderly

**DOI:** 10.3390/ijerph18041996

**Published:** 2021-02-19

**Authors:** Taejun Park, Yun-Sook Jung, Keunbada Son, Yong-Chul Bae, Keun-Bae Song, Atsuo Amano, Youn-Hee Choi

**Affiliations:** 1 Department of Preventive Dentistry, Kyungpook National University School of Dentistry, Daegu 41940, Korea; parktj@gmail.com (T.P.); kbsong@knu.ac.kr (K.-B.S.); 2 Department of Dental Hygiene, Kyungpook National University College of Science & Technology, Sangju 37224, Korea; ysjung0313@knu.ac.kr; 3 Department of Dental Science, Graduate School, Kyungpook National University, Daegu 41940, Korea; sonkeunbada@gmail.com; 4 Department of Anatomy and Neurobiology, School of Dentistry, Kyungpook National University, Daegu 41940, Korea; ycbae@knu.ac.kr; 5 Department of Preventive Dentistry, Osaka University Graduate School of Dentistry, Osaka 565-0871, Japan; amanoa@dent.osaka-u.ac.jp; 6 Institute for Translational Research in Dentistry, Kyungpook National University, Daegu 41940, Korea

**Keywords:** chewing ability, cognitive function, elderly, MMSE, T-scan

## Abstract

Age-related decline in cognitive function is a major challenge in geriatric healthcare. A possible explanation is that the tooth loss or low chewing ability is at cause of cognitive impairment or dementia. The study aimed to investigate the potential relationship between chewing ability and cognitive function in the elderly. A total of 563 participants aged 65 years or over residing in urban and rural areas of South Korea were surveyed. The chewing ability was measured by objectively measurable indications such as the number of remaining teeth, denture status, color-changeable gum, and occlusal balance using T-Scan III^®^. The cognitive function was measured by the Korean version of Mini-Mental State Examination-Dementia Screening (MMSE-DS) and a score of 24 or more (out of 30) indicates a normal cognition, below 23 indicates cognitive impairment. The association between socio-demographic factors, chewing ability factors, and cognitive function demonstrated statistically significant results. When comparing the denture status and chewing ability, the proportion of need denture group had fewer remaining teeth and anterior balanced occlusion. The average number of remaining teeth in anterior balanced occlusion with cognitive impairment was 11.2 compared to posterior balanced occlusion with the normal cognition 19.2. A multiple linear regression analysis declared a significant correlation between number of remaining teeth, denture status, occlusal balance, and cognitive function. Results of the present study revealed objectively measurable indications are suitable for chewing ability assessment and correlated with cognitive function.

## 1. Introduction

The world is aging as the economic and medical growth increased the life expectancy and decreased the birth rate [1]. According to the United Nations’ World Population Ageing Report in 2019, the population of people over the age of 65 across the world is 703 million which accounts for 9% of the total population, and it is reported that the population will continue increasing to 16% by 2050 in which one out of six people will be the elderly [2]. South Korea has already become an aging society as people aged 65 or older accounted for 15.1% of the population in 2019, and it is predicted to be 24.7% in 2030 which will indicate the beginning of super-aged society at an unprecedented speed in the world [3]. As aging progresses, the socio-economic burden is expected to increase due to the decrease in the producing population and increase in the elderly support cost, and the economic growth in the future is expected to slow down [4]. In addition, senile diseases or viral diseases are also expected to increase, and therefore, the interest in prevention and treatment is increasing, and studies on this topic are being actively conducted [5,6,7].

Dementia or cognitive impairment is a disease that occurs mainly in the elderly; the severity of dementia is currently on the rise as the elderly population is rapidly increasing. Dementia usually progresses slowly over long periods of time, leading to various cognitive decline problems due to brain dysfunction, and can even disturb the activities of daily life [8]. Dementia is a neurodegenerative disorder that collectively refers to complex clinical syndromes in which various areas of cognitive function are irreversible and continuously decay [9]. Early symptoms of dementia begin with mild impairments in memory and language function, but dementia can also lead to disorientation to places and people, impaired performance, and psychological behaviors such as depression, delusions, hallucinations, and aggressiveness [10]. The most common type of dementia is Alzheimer’s disease that has various etiologies, such as the beta-amyloid hypothesis and genetic hypothesis, but no fundamental treatment has been developed and only the medications that can alleviate symptoms and delay progression are being used in the clinical practice [11].

According to González-Gross et al., the oral disease may act as a risk factor for senile dementia [12]. Periodontitis, a representative oral disease, leads to tooth loss which causes a decrease in chewing ability. The masticatory exercise has been reported to affect the brain areas of learning and memory as it expands the blood supply to the brain [13]. Evaluating the cognitive functions after feeding only soft food to the experimental animal model, which had posterior teeth or the crown removed, has confirmed that the learning, ability as well as the cognitive function were degraded [14]. There have also been animal experiments which demonstrated that a decrease in chewing ability affects the function and morphology of hippocampal neurons which are essential for the function of learning [15]. In a cross-sectional epidemiologic study that showed similar findings in humans, the elderly with dementia who had cognitive impairment were more likely to have poor oral health or chewing ability [16]. Oral care does not occur appropriately in an individual who has dementia, which results in dental caries and periodontitis from the poor oral condition, and if tooth loss occurs as a result, it leads to a decrease in the chewing ability, which appears to adversely affect the development and progression of cognitive impairment [17].

Loss of functional teeth in oral health means deterioration and weakness in the chewing ability. This can lead to malnutrition and weight loss which cause abnormal systemic health of the body and can cause social alienation or isolation, reducing the quality of life in old age [18]. In addition, reduction in chewing ability can act as a major risk factor for dementia as chronic diseases such as deficiency of essential nutrients, hyperlipidemia, hypertension, and diabetes are known as the causative diseases of dementia [12]. Loss of functional tooth is irreversible, and as much as it adversely affects other teeth, accumulated loss of functional teeth can be used as a last resort marker to reflect the state of oral health [19]. If denture wearers, their status of dentures will affect masticatory. Furthermore, loose or poorly fitting dentures can occlusal pressure decreases, denture stomatitis, and nutritional disorder [20,21]. Therefore, we need to consider the elderly’s oral health condition in combination with the remaining teeth and the fit of dentures.

Many prospective studies have reported a correlation between the number of remaining teeth and cognitive impairment [22]. On the contrary, some studies were not able to identify the correlation [23]. According to a recently published systematic review, an agreement regarding this topic was not reached due to inconsistency in the subjects and methodological differences between the studies [24]. Therefore, it is necessary to conduct studies to identify whether there is a correlation with cognitive function through various methods that can objectively evaluate the chewing ability in the elderly.

The purpose of this study was to check the chewing ability of the elderly through the number of remaining teeth, the use of dentures and occlusions balances, the visual inspection and total color change of masticatory performance evaluating gum, and the occlusal status identified through oral examination in the elderly people over 65 years old who live in urban and rural areas, and to identify a correlation with the cognitive function through a powerful dementia screening tool, Mini-Mental State Examination (MMSE).

## 2. Materials and Methods

### 2.1. Study Participants

This study was conducted on the elderly individuals who are part of Korean Social Life, Health and Aging Project (KSHAP) which was designed based on the National Social Life, Health and Aging Project (NSHAP) hosted by the University of Chicago, USA, as part of an international comparative study on the quality of life and the aging process of the elderly. KSHAP is a panel survey conducted since 2011 to establish integrated indicators on the social, mental, physical, and functional health of the elderly in South Korea [25,26]. The study was approved by the Institutional Review Board of Yonsei University (7001988-201612-SB-306-02) and by the Institutional Review Board of Kyungpook National University Hospital (KNUH 2015-07-007-001).

From February 2018 to July 2018, the purpose of this study was verbally explained to elderly aged 65 years or older, who wished to participate voluntarily, by visiting 22 senior citizen centers in rural villages of K-*myeon* and L-*myeon*, and 14 senior citizen centers of the urban area of N-*gu*. A total of 603 participants provided written consent to participate in the study, and 40 participants who did not respond to the oral examinations or answer the questionnaire were excluded. Therefore, 563 people were selected as the final participants.

Oral examination was performed on the participants by a dentist who was trained by the researcher to examine the number of remaining teeth, use of dentures, chewing ability measured by the changes in the color of masticatory performance evaluating gum, and occlusal balance test. The study was conducted using the interview survey method as the general characteristics and oral health surveys were conducted by three dental hygienists, and the cognitive function test was measured by face-to-face interview with a local nurse in charge of patients with dementia.

### 2.2. Study Participants

#### 2.2.1. Number of Remaining Teeth and the Use of Dentures

For the oral examination, a dentist examined all 32 teeth including the third molar using dental mirrors and World Health Organization (WHO) probes. In accordance with Miller’s tooth movement classification, no movement was recorded as class zero, class one if there was a horizontal movement within 1 mm, class two if there was more than 1 mm horizontal movement, class three if vertical mobility was detected, and teeth in class three were excluded from the total number of remaining teeth in consideration of the functional aspects for mastication [27]. The teeth with only the roots due to implants and crown caries were also excluded. The number of remaining teeth was analyzed by dividing into 4 groups: the edentulous group, 1 to 9 teeth groups, 10 to 19 teeth groups, and more than 20 teeth groups [28].

The use of dentures was analyzed by categorizing the groups into the group which required maxillary or mandibular dentures, partial or full dentures, and natural teeth. Need denture group was restricted into the following categories: fixed implants above three units in the maxillary, mandibular, and maxillary-mandibular (one or both of the jaws), and partial denture or full denture uses.

#### 2.2.2. Occlusal Status Examination

To examine the occlusal balance, the T-scan III^®^ system (Tekscan, Inc., South Boston, MA, USA), a digital occlusal tester, was used in conjunction with Microsoft Windows. Data of higher quality were able to be obtained through this method as it quantitatively depicted the occlusal balance by measuring the objective occlusion force, which is unmeasurable with articulating paper. As a measuring method, the participants were first seated on a chair with head slightly upward above the horizontal plane and bit into centric occlusion. The occlusion sensor pointer of the T-scan III^®^ system was placed between the central incisors of the maxillary to maintain the correct position before measuring the occlusal balance. The recordings of the occlusion were taken by distinguishing the color of occlusion appearing on the graph on the Microsoft Windows screen. The sensitivity was adjusted so the red spots could appear one to three times, and then maintained for approximately two seconds at the central occlusion, and the result of this process repeated three times was recorded. Left and right percentages (%) were recorded to identify unilateral and bilateral occlusion. The images were analyzed in quadrants of the anterior left, anterior right, posterior left, and posterior right to identify the anterior and posterior occlusal balance [29].

### 2.3. Assessment of the Cognitive Function

Mini-Mental Status Examination (MMSE) is the representative dementia screening tool, and the Korean version of MMSE for Dementia Screening (MMSE-DS) was developed by the Ministry of Health and Welfare to standardize dementia screening tools was used [30]. It is used to distinguish between organic and functional mental disorders and is widely used in primary health care fields in South Korea as a cognitive impairment assessment tool. MMSE-DS consists of 30 questions which include assessment, memory registration, memory recall, attention and computation, space-time constructing ability, and language-related skills. Based on the benchmark score, suggested by the developers, of 23 or less, the participants were classified into the cognitive impairment group and normal if the score was 24 or higher [31,32].

### 2.4. Statistical Analysis

The data collected for this study used IBM SPSS (IBM 23.0 for windows, SPSS Inc., Armonk, NY, USA), and the significance level was set to 0.05. Frequency analysis was conducted to confirm the general characteristics of the study participants. Variables related to general characteristics and chewing ability were compared by using *t*-test and one-way ANOVA to identify the relationship with cognitive function. If there was a difference in the mean analyzed by one-way ANOVA, a post hoc test was performed with Tukey, and cross-analysis (Chi-square test, χ^2^) was used to confirm the distribution difference of the participants. Multiple linear regression analysis was conducted to evaluate and predict the relationship between chewing ability and cognitive function, and age, gender, education level, and living arrangement were adjusted and analyzed.

## 3. Results

### 3.1. General Characteristics of Study Participants

The 563 participants ranged from 65 to 97 years old, with an average of 79.4 years of age and 418 women (74.2%) (Table 1). In terms of the level of education, 476 (82.9%) were graduated from elementary school or lower, and 96 (17.1%) received middle school education or higher. The investigation into living arrangements indicated that 212 (37.7%) elderly individuals lived alone, 89 (15.8%) lived with their spouses, and 262 (46.5%) lived with their families excluding, the spouses.

In terms of the number of remaining teeth which, was a variable related to chewing ability, 76 (13.5%) participants were edentulous, 125 (22.2%) had 1 to 9 teeth, 118 (21.0%) had 10 to 19 teeth, and 244 (43.3%) had 20 or more teeth. In the survey on the use of dentures, there were 143 (25.4%) participants who needed but did not have dentures, 168 (29.8%) participants who used partial or full dentures, and 252 (44.8%) participants who had natural functioning teeth. The chewing ability judged through the visual inspection on the color-changeable gum indicated that 95 (16.9%) participants had poor chewing ability (1–3 points) and 468 (83.1%) participants had good chewing ability (4–5 points). The occlusion balance identified using the T-scan III^®^ system showed that 288 (51.2%) participants had unilateral balanced occlusion and 275 (48.8%) participants had bilateral balanced occlusion. When the occlusion balance of the anterior and posterior was measured, 343 (60.9%) participants showed strong anterior balanced occlusion, and 220 patients (39.1%) showed strong posterior balanced occlusion.

MMSE-DS showed an average score of 24.4. The 210 participants scored less than 23 which classified them cognitively impaired, and 353 (62.7%) participants scored 24 or higher as normal.

### 3.2. Differences in the Cognitive Function according to General Characteristics

When the average score of MMSE-DS was compared according to the general characteristics, the proportion of cognitive impairment was higher with older age (*p* < 0.001) as participants who were younger than 80 years of age had a score of 25.44 ± 3.17 compared with participants older than 80 years of age scored 23.41 ± 4.08 (Table 1). The average MMSE-DS score for the elderly men was 25.76 ± 3.17 and women 23.94 ± 3.89 which demonstrated that the proportion of cognitive impairment was higher in the elderly women (*p* < 0.001). Those who received education higher than middle school had lower MMSE-DS scores than those who graduated from elementary school or received less than elementary school level education (*p* < 0.001), and the percentage of cognitive impairment was significantly higher (*p* < 0.001) in the latter group. The average score of MMSE-DS according to the living arrangement was 23.80 ± 3.85 in the participants who live alone, 22.87 ± 4.50 who live with family members excluding the spouse, and 25.42 ± 3.80 who live with their spouse, and the rate of cognitive impairment was high in the participants who live alone (*p* < 0.001).

### 3.3. Differences in Cognitive Functions according to the Mastication Ability

When the mean scores of MMSE-DS were compared by dividing the number of remaining teeth into four groups, the score was 24.11 ± 3.77 in the edentulous, 23.40 ± 4.16 in 1 to 9 teeth, 24.24 ± 4.05 in 10 to 19, and 25.10 ± 3.35 in 20 or more teeth group (*p* < 0.001) (Table 1). In terms of the use of dentures, the average MMSE-DS score was 23.80 ± 3.93 for need denture, 24.05 ± 4.07 for partial or full denture, and 25.02 ± 3.46 for natural teeth, which was statistically significant (*p* < 0.001). Visual inspection of color-changeable gum showed the score of MMSE-DS in the poor group was 23.20 ± 3.69, lower than the good group 24.65 ± 3.78 (*p* < *0*.001). Following the anterior and posterior balanced occlusion measured by the T-scan III^®^ system, the average MMSE-DS was higher in the group with molar occlusion (*p* < 0.001).

### 3.4. Relationship between the Variables Associated with Chewing Ability and the Denture Status

When comparing the denture status and the number of remaining teeth, need denture with 10 to 19 teeth (54.5%), as the group was expected to decrease in the chewing ability due to the extraction of functional teeth (Table 2). The proportion of participants who wore dentures was highest in the group which had 1 to 9 teeth (50.0%), and the group with more than 20 teeth (85.3%) had natural teeth. When comparing the occlusal balance and denture status, the proportion of individuals who need dentures was higher (*p* < 0.001) in the group with lower chewing ability which used the anterior balanced occlusion (77.6%) than in the group with higher chewing ability which used the posterior balanced occlusion (22.4%).

### 3.5. Relationship between the Presence of Cognitive Impairment and the Number of Remaining Teeth according to Anterior and Posterior Balanced Occlusion

The average number of remaining teeth in the anterior balanced occlusion participants with cognitive impairment was 11.2, which was statistically significantly lower than the average of 17.2 teeth in the posterior balanced occlusion (*p* < 0.001) (Table 3). On the contrary, the average number of remaining teeth in the posterior balanced occlusion with normal cognitive function was 19.2, which was statistically higher (*p* < 0.001) than the average of 14.0 teeth in the anterior balanced occlusion.

### 3.6. Chewing Ability and the Cognitive Function-Multiple Linear Regression Analysis

The results confirm the relationship between the objectively measured chewing ability variables and cognitive function using multiple linear regression analysis (Table 4). There was a significant correlation between the number of remaining teeth, the denture status, the visual inspection of color-changeable gum, and the anterior and posterior balanced occlusion, with the MMSE-DS scores with no adjustment in the variables (*p* < 0.05). After adjusting the general characteristic variables such as age, gender, education level, and living arrangement, there were statistically significant correlations still evident between the number of remaining teeth, the denture status, the visual inspection of color-changeable gum, and the anterior and posterior balanced occlusion, with MMSE-DS scores (*p* < 0.05).

## 4. Discussion

The chewing ability is the main function of the oral cavity and is essential for humans to receive the nutrients necessary for life. In particular, nutrition intake in the elderly is even more crucial as it is closely related to extending their lives and maintaining health. Many factors influence the chewing ability. Van der Bilt stated that chewing ability is related to dentition including the number of teeth and the anatomical structures and that the number of teeth is the single factor that can exert the most influence [33]. Deterioration of chewing ability in the elderly was caused by tooth loss, the chewing ability and deterioration of saliva secretion, and the effects are known to cause not only malnutrition, but also daily living performance deterioration and cognitive impairment [34,35]. In addition, posterior teeth occlusion was independently associated with functional dependence in the elderly [36,37].

This study used a variety of methodologies to objectively assess the chewing ability of 563 people aged 65 or older who live in urban and rural areas to analyses the effect of chewing ability on cognitive function.

According to this study results, the higher the number of remaining teeth and the higher the MMSE-DS score (ß = 0.065, *p* < 0.001). This result means that as in previous studies, indicating that the number of teeth and masticatory ability of the elderly are related to cognitive impairment [12,13,14,38]. As for denture status, the MMSE-DS score of unnecessary subjects was higher than those who needed dentures (ß = 0.036, *p* = 0.005), and in occlusal balance, the subjects with better posterior occlusion than anterior occlusion showed higher MMSE-DS score (ß = 0.113, *p* = 0.006). The results also demonstrated similar patterns as differences in another study about better the dentures were and more occlusion force of posterior teeth with better cognitive ability [36,37,39].

Shibuya suggested that chewing ability is positively correlated with cognitive function [40]. Paganini-Hill et al. found similar results to this study which identified that chewing with dentures induces a higher risk of dementia than chewing with natural teeth [41]. Loss of teeth is a risk factor that leads to reduction in the cognitive function from the decreased occlusion and mastication. Avlund et al. reported that individuals with fewer remaining teeth had lower cognitive function [42], and Chang et al. reported that cognitive function was low when individuals had inappropriate dentures [43]. Therefore, it can be seen that the number of remaining teeth and the occlusal balance of the dentures influence the cognitive function of the elderly.

The limitations of this study are as follows: First, this was a cross-sectional study which cannot suggest the cause-and-effect relationship. Second, there are limitations in generalizing the results as the investigation was conducted on selected senior citizen centers based on convenient sampling. Third, it is difficult to compare the gender characteristics as the number of elderly men was smaller than that of elderly women. However, there are gender differences in the overall proportion of the elderly as women have a longer life expectancy than men, and it is known that a actively participate in social activities, such as visiting the senior citizen center, as this study investigated. Therefore, there is a need to analyze gender-related variables in detail by evenly allotting the distribution of men and women for future studies. Fourth, the participants’ education levels were biased as the highest level of educational attainment for the majority of the participants was at or below the elementary school. However, this bias in the educational level does not deviate significantly from the representativeness of the elderly as the majority of the elderly aged 80 years or older in South Korea were educated to or below the elementary school level.

In spite of such limitations, this study has significance as it quantified the variables which influence the chewing ability of the elderly using objective methods and confirmed their association with cognitive function. Furthermore, it is thought that the direction of research on the relationship between oral health and cognitive function can be suggested through follow-up studies which include socio-statistical analysis. It is anticipated that the results of this study will improve the chewing ability of the elderly more effectively to prevent cognitive impairment.

## 5. Conclusions

In this study, that the impact on cognitive function was analyzed using various methodologies that can objectively evaluate the chewing ability of the 563 participants aged 65 or older living in urban and rural areas of South Korea. According to the results, there was a significant correlation between cognitive function and the number of remaining teeth, the presence of dentures, and the anterior/posterior occlusal patterns. This study suggests the possibility of a significant relationship between the chewing ability and cognitive function.

## Figures and Tables

**Table 1:** Cognitive function with socio-demographic and chewing ability.

		Cognitive Impairment				
		MMSE-DS Score		Yes (*n* = 210)	No (*n* = 353)	
	*N* (%)	Mean ± SD	*p*-Value	*N* (%)	*N* (%)	*p*-Value
Age			<0.001 *			<0.001 **
<80	277 (49.2)	25.44 ± 3.17		69 (32.9)	208 (58.9)	
≥80	286 (50.8)	23.41 ± 4.08		141 (67.1)	145 (41.1)	
Gender			<0.001 *			<0.001 **
Male	145 (25.8)	25.76 ± 3.17		31 (14.8)	114 (32.3)	
Female	418 (74.2)	23.94 ± 3.89		179 (85.2)	239 (67.7)	
Education			<0.001 *			<0.001 **
≤Elementary	467 (82.9)	23.97 ± 3.86		196 (93.3)	271 (76.8)	
>Elementary	96 (17.1)	26.51 ± 2.63		14 (6.7)	82 (23.2)	
Living arrangement			<0.001 *			<0.001 **
Alone	212 (37.7)	22.87 ± 4.50		98 (46.7)	114 (32.3)	
With spouse	89 (15.8)	23.80 ± 3.85		50 (23.8)	39 (11.1)	
With family	262 (46.5)	25.42 ± 3.80		62 (29.5)	200 (56.6)	
Denture status			0.002 *			0.006 **
Need denture	143 (25.4)	23.80 ± 3.93		60 (28.6)	83 (23.5)	
Denture wearer	168 (29.8)	24.05 ± 4.07		74 (35.2)	94 (26.6)	
Natural teeth	252 (44.8)	25.02 ± 3.46		76 (36.2)	176 (49.9)	
Number of remaining teeth			<0.001 *			0.002 **
0 **	76 (13.5)	24.11 ± 3.77		30 (14.3)	46 (13.0)	
1–9	125 (22.2)	23.40 ± 4.16		59 (28.1)	66 (18.7)	
10–19	118 (21.0)	24.24 ± 4.05		51 (24.3)	67 (19.0)	
≥20	244 (43.3)	25.10 ± 3.35		70 (33.3)	174 (49.3)	
Color-changeable gum			0.001 *			0.003 **
Poor (1–3)	95 (16.9)	23.20 ± 3.69		48 (22.9)	47 (13.3)	
Good (4–5)	468 (83.1)	24.65 ± 3.78		162 (77.1)	306 (86.7)	
Occlusion balance I			0.962			0.331
Unilateral	288 (51.2)	24.40 ± 3.90		113 (53.8)	175 (49.6)	
Bilateral	275 (48.8)	24.41 ± 3.70		97 (46.2)	178 (50.4)	
Occlusion balance II			<0.001 *			0.007 **
Anterior	343 (60.9)	23.96 ± 3.94		143 (68.1)	200 (56.7)	
Posterior	220 (39.1)	25.10 ± 3.47		67 (31.9)	153 (43.3)	

Occlusion balance I: Bilateral balanced occlusion. Occlusion balance II: Anterior and posterior balanced occlusion. * *p* < 0.05 by one-way ANOVA or *t*-test. ** *p* < 0.05 by χ^2^-test.

**Table 2:** Chewing ability according to denture status.

	Denture Status			
	Need Denture (*N* = 143)	Denture Wearer (*N* = 168)	Natural Teeth (*N* = 252)	*p*-Value *
	*N* (%)	*N* (%)	*N* (%)	
Number of remaining teeth				<0.001
0	0 (0.0)	76 (45.2)	0 (0.0)	
1–9	39 (27.3)	84 (50.0)	2 (0.8)	
10–19	78 (54.5)	5 (3.0)	35 (13.9)	
≥20	26 (18.2)	3 (1.8)	215 (85.3)	
Color-changeable gum				<0.001
Poor (1–3)	17 (11.9)	67 (39.9)	11 (4.4)	
Good (4–5)	126 (88.1)	101 (60.1)	241 (95.6)	
Occlusion balance I				0.980
Unilateral	73 (51.0)	85 (50.6)	130 (51.6)	
Bilateral	70 (49.0)	83 (49.4)	122 (48.8)	
Occlusion balance II				<0.001
Anterior	111 (77.6)	116 (59.0)	116 (46.0)	
Posterior	32 (22.4)	52 (31.0)	136 (54.0)	

* One-way ANOVA or *t*-test. Occlusion balance I: Bilateral balanced occlusion. Occlusion balance II: Anterior and posterior balanced occlusion.

**Table 3:** Anterior and posterior balanced occlusion according to cognitive function and number of remaining teeth.

	Cognitive Impairment		
	Yes (*N* = 210)	No (*N* = 353)	*p*-Value *
	Number of Remaining Teeth		
	*N* ± SD	*N* ± SD	
Occlusion balance II			<0.001
Anterior	11.2 ± 9.3	14.0 ± 9.6	
Posterior	17.2 ± 10.8	19.2 ± 10.9	

* Chi-square test, χ^2^. Occlusion balance II: Anterior and posterior balanced occlusion.

**Table 4:** Multiple linear regression analysis of chewing ability and cognitive function.

		MMSE-DS Score									
		Model I (Crude)			Model II			Model III			
	Reference	B	ß	*p*-Value	B	ß	*p*-Value	B	ß	*p*-Value	VIF
Number of remaining teeth		0.399	0.279	<0.001	0.290	0.195	<0.001	0.184	0.065	<0.001	1.632
Denture status	Need denture	0.294	0.203	<0.001	0.226	0.182	0.003	0.122	0.036	0.005	1.357
Color-changeable gum	Poor	0.996	0.098	0.037	0.788	0.078	0.085	0.755	0.075	0.096	1.309
Occlusion balance I	Unilateral	−0.065	−0.009	0.837	−0.218	−0.029	0.471	−0.177	−0.023	0.554	1.023
Occlusion balance II	Anterior	0.989	0.127	0.004	0.894	0.115	0.006	0.881	0.113	0.006	1.114
Age	<80				1.771	0.233	<0.001	1.543	0.203	<0.001	1.115
Gender	Male				−1.605	−0.185	<0.001	−0.753	−0.087	0.047	1.472
Education	≤Elementary							1.548	0.153	0.001	1.296
Living arrangement	Alone							0.320	0.077	0.048	1.251

*p*-values by multiple linear regression. B: Unstandardized beta; ß: Standardized beta; VIF: Variance Inflation Factors. Model I: Unadjusted model (Crude). Model II: Model I + Age and gender adjusted model. Model III: Model I + Age, gender, education, and living arrangement adjusted model.

## Data Availability

Data are contained within the article.
